# Efficacy of pulmonary rehabilitation combined with minimal-dose adjunctive TCM (Shen Yi Capsules and Hua Chan Su) in improving postoperative outcomes for non-small cell lung cancer: a randomized controlled trial

**DOI:** 10.3389/fmed.2026.1761427

**Published:** 2026-05-06

**Authors:** Ge Chunyan, Wang Fengshuang, Li Mei, Chen Zhiqun, Han Dongmei

**Affiliations:** Department of Organ Rehabilitation, Shanghai First Rehabilitation Hospital, Shanghai, China

**Keywords:** breathing techniques, St. George's Respiratory Questionnaire (SGRQ), idiopathic pulmonary fibrosis, interstitial lung disease, pulmonary rehabilitation

## Abstract

**Background:**

Interstitial lung disease (ILD) is a chronic respiratory disease that is characterized by progressive pulmonary fibrosis, impaired gas exchange, and reduced exercise tolerance.

**Objective:**

The present investigation aimed to evaluate the efficacy of a systematically organized pulmonary rehabilitation regimen in improving functional capacity and health related quality of life of individuals affected by ILD.

**Method:**

This study assessed the effects of a structured 3-month PR program conducted at Shanghai First Rehabilitation Hospital, China, using pre- and post-intervention assessments comprising spirometry, the St. George's Respiratory Questionnaire (SGRQ), and the six-minute walk test (6MWT). This study assessed the effects of a structured 3-month pulmonary rehabilitation (PR) program conducted at Shanghai First Rehabilitation Hospital, China, using pre- and post-intervention assessments. These assessments included spirometry, the St. George's Respiratory Questionnaire (SGRQ), and the 6-minute walk test (6MWT). Alongside the PR program, patients received minimal-dose adjunctive Traditional Chinese Medicine (TCM), specifically the administration of Shen Yi Capsules and Hua Chan Su, which were used to investigate their combined impact on improving postoperative outcomes for non-small cell lung cancer (NSCLC) patients.

**Results:**

Upon the completion of the pulmonary rehabilitation programme, participants showed significant improvements indicating by statistical significance of the decrease in St. George's Respiratory Questionnaire (SGRQ) scores (*p* = 0.001) and the average improvement of the six-minute walk distance by 42.1 m. Program adherence was favorable (90 %), although participants faced transportation, financial, and physical barriers, as well as poor patient awareness.

**Conclusion:**

The results suggest that a customized PR program is both safe and effective in improving health-related quality of life (HRQOL) and exercise capacity in patients with ILD, underscoring the importance of developing strategies to mitigate barriers to accessing these programs and thereby maximizing benefit for patients.

## 1 Introduction

Interstitial lung disease, or ILD, is a heterogeneous group of chronic pulmonary disorders characterized by progressive fibrosis, impaired gas exchange and reduced functional capacity (1). Individuals diagnosed with ILD often experience dyspnea, constant fatigue, as well as restrictions on routine activities, significantly reduced health-related quality of life (HRQOL). Pulmonary rehabilitation (PR) has therefore become an important non-pharmacological intervention approach, aiming to improves exercise tolerance, relieve symptoms, and increase the overall quality of life for the patients who suffer from chronic disorders of the respiratory system. These issues are particularly pronounced in patients with preexisting pulmonary diseases, postoperative fibrosis, or treatment-related lung injury all of which resemble interstitial lung disease (ILD) in their restrictive ventilatory defects and impaired gas exchange (2).

**Figure 1:**
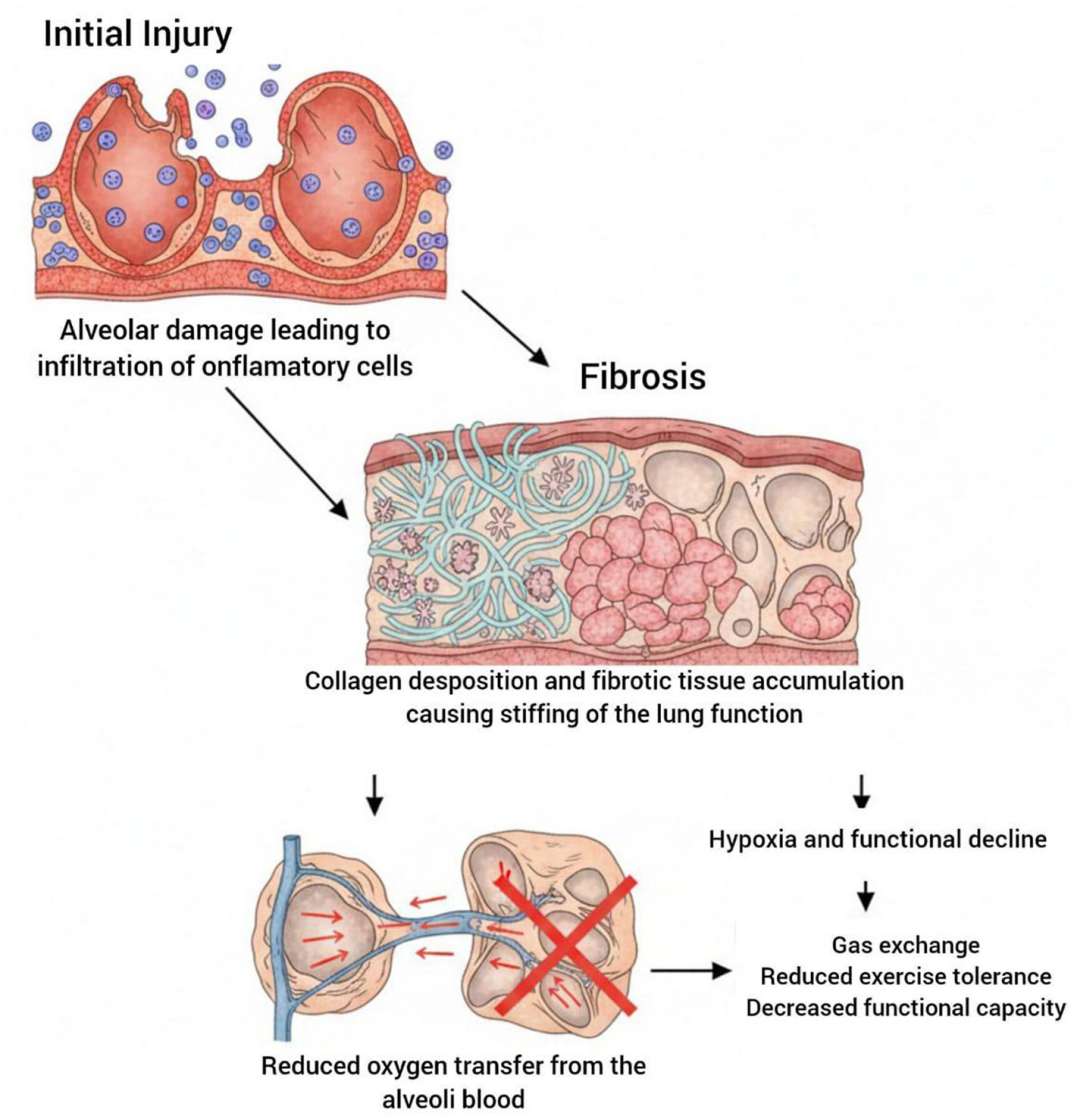
Conceptual illustration of key pathophysiological features of interstitial lung disease.

Pulmonary rehabilitation programs that combine exercise training, breathing techniques, patient education and psychosocial support have shown significant improvement in functional capacity and quality of life in patients diagnosed with chronic respiratory disease (3). Figure 1 illustrates the pathological mechanisms of ILD, including fibrosis, impaired gas exchange, and reduced exercise tolerance.

To further clarify the argument, the management of interstitial lung diseases (ILDs)—a heterogeneous group characterized by irreversible parenchymal damage and significant reductions in functional capacity and HRQOL demonstrates the critical role of non-pharmacological interventions. Individuals with interstitial lung disease often struggle with persistent dyspnea, significant fatigue, and significant psychological distress which severely limits routine activities and impacts health-related quality of life (4). Because pharmacologic interventions have only limited effects on symptoms and function, maximizing physical capacity and quality of life through non-pharmacological means is increasingly relevant in clinical practice. Pulmonary rehabilitation (PR) has become a pillar of care for chronic obstructive pulmonary disease (COPD). Still, its specific value in ILD remains under active investigation—a point central to the current argument. While new data suggest that PR may relieve symptoms and improve exercise performance in ILD, these benefits are typically more limited and short-lived than those in COPD. This difference underscores the need to tailor rehabilitation based on differing disease mechanisms and progression in restrictive vs. obstructive lung diseases (5).

The main argument is that more rigorous studies are required to define how ILD subtype and baseline disease severity affect PR outcomes. Current reviews show that, although PR benefits most ILD subtypes, the degree and durability of improvement may be reduced in idiopathic pulmonary fibrosis (IPF) due to greater physiological impairment. Addressing this critical gap would enable better patient selection and more effective rehabilitation strategies for ILD treatment (6).

Despite progress in pharmacotherapy, especially the antifibrotic agents for fibrotic ILDs, no curative treatment exists. As a result, the international guidelines have increasingly focused on the use of PR as an important supportive therapy for ILD management (7). However, these same guidelines highlight major uncertainties, such as when to enroll children, the optimal role of programs, and the extent to which improvements are sustained after program completion (8). Empirical evidence related to the efficacy of pulmonary rehabilitation in persons with an interstitial lung disease diagnosis remains lacking in many clinical settings, particularly smaller rehabilitation facilities in which structured interventions of PR are still emerging (9). In addition, patient-reported outcomes, such as the St. George's Respiratory Questionnaire (SGRQ), have rarely been included in clinical studies on ILD, limiting understanding of the influence of PR on patients' perceived quality of life over time (10, 11).

Patient-Reported Outcome Measures (PROMs) are a key way to provide an important perspective on the burden of disease and the impact of treatment, in addition to physiological assessment (12, 13). The SGRQ, a widely validated respiratory-specific PROM, has been extensively used in the study of patients with chronic obstructive pulmonary disease (COPD) but is yet to be underutilized in patients with ILD, especially in low- and middle-income countries (14, 15). Given the lack of high-quality evidence and the contextual nature of healthcare access, there is a critical need for robust, region-specific data on the effectiveness of PR in ILD, using standardized PROMs such as the SGRQ (16).

The objective of the present study is to evaluate the effectiveness of a structured pulmonary rehabilitation program in improving the health-related quality of life of individuals with ILD, using the St. George's Respiratory Questionnaire as the primary outcome measure. Pulmonary rehabilitation has proven to improve exercise tolerance, lessen dysphagia, and significantly improve patient-reported outcomes of individuals with chronic respiratory diseases, such as interstitial lung disease (ILD). Nevertheless, region-specific evidence to define its efficacy is still sparse, particularly in developing healthcare settings.

To what extent does a carefully designed pulmonary rehabilitation program improve St. George's Respiratory Questionnaire (SGRQ) outcomes and functional exercise capacity in those diagnosed with interstitial lung disease? While the larger clinical context is the pulmonary sequelae following resection of lung cancer, the current investigation focuses squarely on the assessment of the efficacy of pulmonary rehabilitation in the interstitial lung disease cohort.

### 1.1 Pulmonary rehabilitation in interstitial lung disease

Pulmonary rehabilitation (PR) is a well-known form of non-pharmacologic treatment that aims at improving exercise capacity, decreasing dyspnea, and increasing quality of life in patients with chronic respiratory diseases (17). In most cases PR encompasses organized physical workouts, breathing exercises, patient educative sessions, and behavioral management solutions, which enhances the functional capacity and better symptoms management in this patient group.

Although the evidence base of PR in chronic obstructive pulmonary disease is still voluminous, growing evidence now provide that patients with interstitial lung disease can also receive the benefit of carefully planned rehabilitation program. Gains in six-minute walk distance (6MWD), decreases in symptom burden, and positive changes in measures of health-related quality of life have persistently been reported in ILD populations after PR interventions (18).

Although there are substantial amounts of evidence, the data are still sparse, particularly when based on small clinical cohorts or used in actual rehabilitation settings. Subsequent studies are therefore justified to determine the practicability and the future potential benefits of pulmonary rehabilitation program to the patients with interstitial lung disease (19).

PR is a multidisciplinary treatment involving exercise training, patient education and behavioral support all of which is designed to improve the physical and psychological wellbeing of individuals with chronic respiratory diseases (20). Although PR has long been used in the management of chronic obstructive pulmonary disease (COPD), it has recently been increasingly applied to patients with interstitial lung disease (ILD) (2). ILD, with one of its types being idiopathic pulmonary fibrosis (IPF), is a disease that involves progressive fibrotic remodeling of the lung parenchyma, resulting in restrictive ventilatory defects, compromised gas exchange, reduced diffusion ability, exercise intolerance, and critical deterioration of health-related quality of life (HRQOL) (12). It is interesting to note that the physiologic sequelae seen in patients with ILD are remarkably reminiscent of functional limitations observed in other chronic respiratory conditions, implying PR has the potential to provide these two patient groups with comparable functional and symptomatic advantages (21).

**Figure 2:**
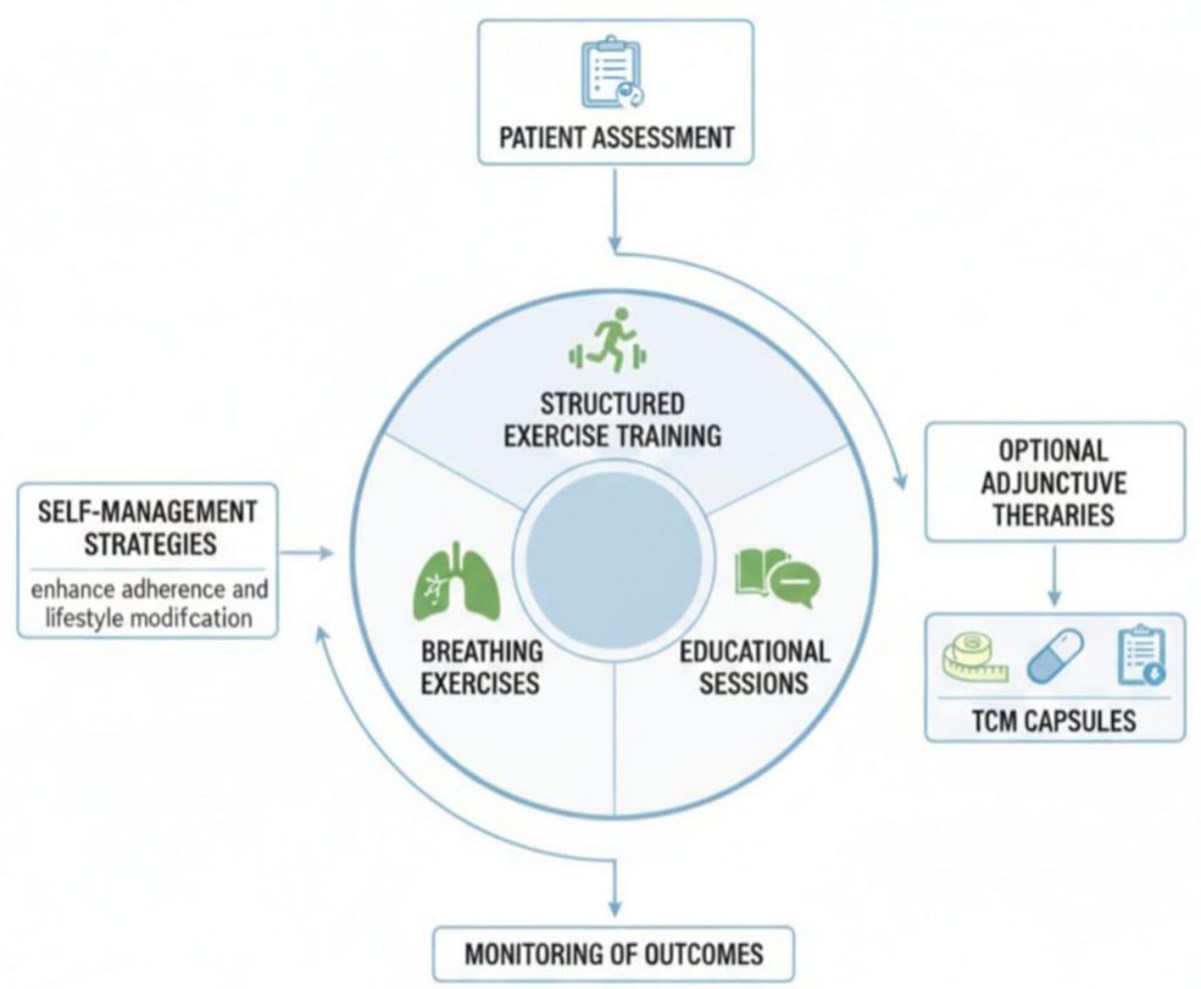
Conceptual framework illustrating the general components of pulmonary rehabilitation programs for patients with interstitial lung disease.

New findings show that organized PR initiatives yield clinically significant improvements in functional capacity, dyspnea severity, and HRQOL in patients with ILD. For example, improvements of 30 to 60 meters in the six-minute walk distance (6MWD) can be compared with the minimal difference in clinical importance of functional performance, which is established as a minimum (22). In the same way, validated measures such as the St. George Respiratory Questionnaire (SGRQ) consistently identify significant post-rehabilitation changes in symptom load, activity restrictions, and overall perceived health (23). Notably, PR has been demonstrated to be effective and safe even in patients with severe pulmonary dysfunction, highlighting its universal nature and usefulness regardless of disease severity. Figure 2 depicts the core components of PR pulmonary rehabilitation programs for patients with interstitial lung disease (24). These barriers show that, to maximize effectiveness and adoption, PR programs need to be more culturally specific, accessible, and affordable.

## 2 Methodology

### 2.1 Study design

**Figure 3:**
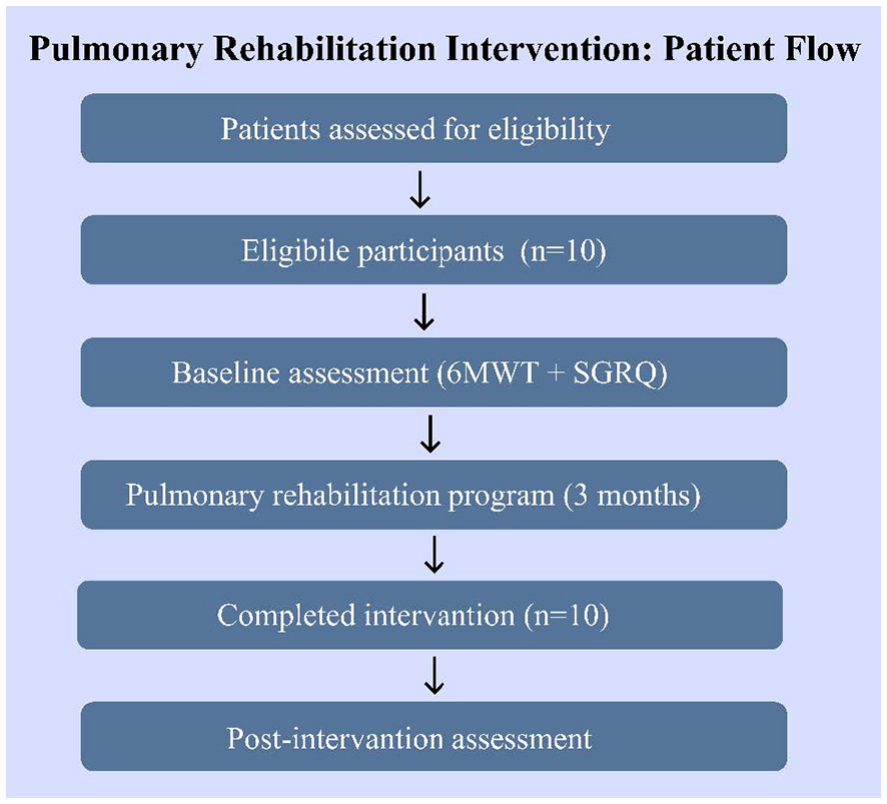
Participant flow diagram illustrating recruitment, enrollment, pulmonary rehabilitation participation, and outcome assessment of study participants.

The present study used a quasi-experimental pre-post intervention design to test the effectiveness of a structured pulmonary rehabilitation (PR) programme in a group of patients with interstitial lung disease (ILD) as shown in Figure 3. The study was conducted in the Shanghai First Rehabilitation Hospital which is a tertiary rehabilitation facility with expertise in respiratory care and the study is a clinical study designed to investigate the potential benefits of PR in a small group of patients with ILD. Adopting a single-group quasi-experimental design, participants were their own controls, thus, allowing intra-subject comparisons between the timeframes of the study.

The results were also measured at two important points at the time when the pulmonary rehabilitation was going to start and at the end of the 3-month period of intervention.

### 2.2 Participants and eligibility criteria

The cohort was selected among those who were included in the respiratory rehabilitation unit. Inclusion criteria were patients diagnosed with interstitial lung disease confirmed by clinical examination and radiographic studies and in a stable clinical condition and able to participate in a structured rehabilitation intervention. The exclusion criteria included patients having unstable cardiovascular pathology, a severe mobility restriction that would make it impossible to participate in exercise training, or the acute respiratory infection during the enrolment date. Ten subjects who met the pre-specified eligibility criteria were recruited into the study and they were able to complete the pulmonary rehabilitation programme.

#### 2.2.1 Sample size

The study was planned as a exploratory study and hence no formal statistical power analysis was performed before the study participants were enrolled. The general objective of the research was to determine the feasibility of the use of structured pulmonary rehabilitation programme and to acquire initial estimations of functional capacity and quality-of-life outcomes in those with the interstitial lung disease.

Given the exploratory nature of the research and the small number of qualified respondents that can be found within the specified period of recruitment, a small convenience sample of 10 participants was included. This size is typical of studies that aim to determine feasibility, test the operations of the tool, and provide raw information about a new drug to be used to inform the full, larger-scale, clinical trials design, and sample-size calculation.

#### 2.2.2 Pulmonary rehabilitation program

The tri-month period was that of the structured pulmonary rehabilitation programme, which included supervision-based sessions covering the focus on exercise training, respiratory mechanics, patient education, and psychosocial support. The physical exercise programme involved aerobic conditioning, pulmonary rehabilitation exercises, and low-intensity resistance exercises that were all formulated to enhance cardiopulmonary endurance and functional capacity in general. All the components were provided in an orderly fashion to optimize patient outcomes.

A multidisciplinary rehabilitation team consisting of respiratory therapists, physiotherapists and rehabilitation physicians working in Shanghai First Rehabilitation Hospital coordinated the intervention. The individual sessions were carefully prepared according to the initial functional status of the patient and the development of the clinical picture such that the intensity and content of the therapy were adjusted to the individual to each participant.

The participants attended supervised sessions several times per week and had homework to fulfill breathing and physical exercise programmes recommended at home. Compliance was strictly observed by monitoring the attendance record and frequent check up by the rehabilitation team. The patients were continuously encouraged, counseled, and reinforced in educational aspects to ensure that they remained engaged in the programme.

#### 2.2.3 Adherence monitoring

Adherence to pulmonary rehabilitation programme in the study was monitored systematically with careful attendance recordings and regular clinical follow ups to the participants by the rehabilitation team. They were advised to continue with exercises of breathing and exercise, as well as, to attend scheduled rehabilitation sessions at the home environment. Educational support and counseling were provided to improve participation and compliance to the programme.

#### 2.2.4 Intervention fidelity and adherence monitoring

Administration of the rehabilitation programme by the presence of a multidisciplinary rehabilitation team consisting of respiratory physicians, physiotherapists and rehabilitation therapists ensured intervention fidelity. The observed consistency in the methodology of the therapeutic delivery was achieved by strict adherence to standardized rehabilitation protocols in the supervised sessions hence eliminating variability in the therapeutic delivery.

The compliance of the participants to the pulmonary rehabilitation programme was monitored systematically through detailed attendance records and quarterly follow-up evaluations performed by the rehabilitation team. Patients were also actively supported to come to regular rehab classes and practice the breathing exercises and physical activities given during domiciliary schedules.

#### 2.2.5 Outcome measures

The functional capacity and health-related quality of life were measured using validated outcome measures at baseline and after the rehabilitation intervention was completed. St. George's Respiratory Questionnaire (SGRQ) was scored as per the set scoring algorithm and produced domain and total scores of 0 to 100 with the higher the response, the poor the health status. The main efficacy outcomes included the Six-Minute Walk Test (6MWT) as an objective measure of functional exercise capacities and SGRQ as a subjective measure of the health-related quality of life.

The primary outcome measures included:

- Six-Minute Walk Test (6MWT) to evaluate functional exercise capacity
- St. George's Respiratory Questionnaire (SGRQ) to assess health-related quality of life

### 2.3 Participant flow description

Participant recruitment and study progression were carried out in accordance with a rigorous, methodologically sound, sequential protocol. Individuals with interstitial lung disease presenting at the rehabilitation clinic underwent a strict eligibility screening. Prior to the initiation of the pulmonary rehabilitation regimen, eligible participants were systematically evaluated at baseline. Upon completion of the 3-month intervention participants were re-evaluated using the same functional and quality of life instruments to establish comparability and validity of outcome measures.

### 2.4 Statistical analysis

Data were analyzed with paired sample statistical tests to compare baseline and post intervention outcomes. Continuous variables were presented as the mean plus or minus standard deviation (SD). Paired *t*-tests were used to determine changes in the six-minute walk test (6MWT) and Saint George's Respiratory Questionnaire (SGRQ) scores between pre- and post-intervention measurements. A *p-value* of < 0.05 was deemed to be indicative of statistical significance.

## 3 Results

### 3.1 Participant characteristics

Ten respondents who met the eligibility criteria were included in the analysis. Table 1 summarizes baseline demographic and clinical characteristics. The cohort had a mean age of 54 years with a standard deviation of 10 years. This reflects a middle-aged group typically vulnerable to chronic interstitial lung diseases. The initial recruitment was gender-balanced, but females made up a slightly larger sample (60%) than men (40%). This distribution generally represents the population in the region.

**Table 1:** Baseline demographic characteristics of the study participants (n = 10).

Characteristic	Value
Age (years)	54 ± 10
Gender	Male – 4 (40%), Female – 6 (60%)
Education	Illiterate – 2 (20%), Primary – 5 (50%), High school – 2 (20%), Graduate/Postgraduate – 1 (10%)
Occupation	Low-risk – 6 (60%), Moderate-risk – 2 (20%), High-risk – 2 (20%)
Marital status	Married – 7 (70%), Widowed – 3 (30%)

There was notable variation in education among participants. Half of the cohort (50%) had primary-level education, and 20% reported no education. Only 1 participant (10%) had graduated or postgraduate education, indicating a low overall educational background. Occupational exposure patterns showed that 60% of participants were employed in low-risk jobs. The remaining 40% reported moderate to high occupational exposure risk, which could have contributed to the onset or progression of the disease. Most subjects were married (70%), while 30% were widowed at enrollment. These marital status differences might affect adherence to psychosocial support and rehabilitation. All participants were recruited by the respiratory rehabilitation clinic of the Shanghai First Rehabilitation Hospital, and were receiving routine clinical follow-up at the same institution.

### 3.2 Distribution of ILD subtypes

Table 2 presents the clinical spectrum of interstitial lung disease in the study population. Idiopathic Interstitial Pneumonias (IIPs) accounted for 60 percent of participants. Within this category, the most common phenotype was usual interstitial pneumonia (UIP), which included idiopathic pulmonary fibrosis (IPF-UIP) and non-specific interstitial pneumonia (NSIP-ILD). This distribution aligns with previous data showing that UIP is the most common IIP subtype in tertiary care.

**Table 2:** Distribution of Interstitial Lung Disease (ILD) subtypes among participants (n = 10).

Disease category	Frequency	Percentage
Idiopathic Interstitial Pneumonia (IIP)	6	60.0
Granulomatous ILD	3	30.0
Connective tissue disease–associated ILD	1	10.0

Granulomatous lung diseases accounted for 30% of the cohort, underscoring the ongoing burden of inflammatory and immune-mediated lung diseases in developing healthcare systems. One participant (10%) showed etiological heterogeneity of ILD associated with connective tissue disease. The inclusion of combined pulmonary fibrosis and emphysema (CPFE) cases underscores the diagnostic and classification challenges in the IIP spectrum.

### 3.3 Functional capacity and quality of life outcomes

Paired-sample analyses of baseline and post-intervention outcomes showed statistically significant improvement in functional exercise capacity and health-related quality of life after the completion of the 3-month pulmonary rehabilitation program (Table 3). Participants showed improvement of their Six-Minute Walk Test (6MWT) distance by an average difference of 42.1 (SD = 25.36) meters after the intervention. This amelioration shows a substantial increase in physical functioning and exercise tolerance.

**Table 3:** Pre- and post-intervention functional and quality-of-life outcomes.

Outcome measure	Baseline Mean ±SD	Post-intervention Mean ±SD	Mean difference	95% confidence interval	*p-value*
Six-Minute Walk Test (6MWT)	342.5 ± 65.4	384.6 ± 67.8	42.1	22.3 to 61.9	0.001
SGRQ symptom score	56.4 ± 12.6	44.0 ± 11.5	−12.4	−19.6 to −5.2	0.001
SGRQ total score	58.3 ± 14.2	42.4 ± 13.8	−15.9	−24.3 to −7.5	0.001

On the same note, there were also significant positive changes in health-related quality-of-life metrics. In the present study, SGRQ Symptom score showed a statistically significant improvement after the pulmonary rehabilitation program with mean decrement of 12.40 points (*p* = 0.001) thus supporting a clinically meaningful reduction in the burden of symptoms. This result is representative of an improved perception of symptoms post-rehabilitation. Furthermore, the aggregate SGRQ score decreased by an average of 15.87 points, indicating a significant improvement in disease associated quality of life.

**Figure 4:**
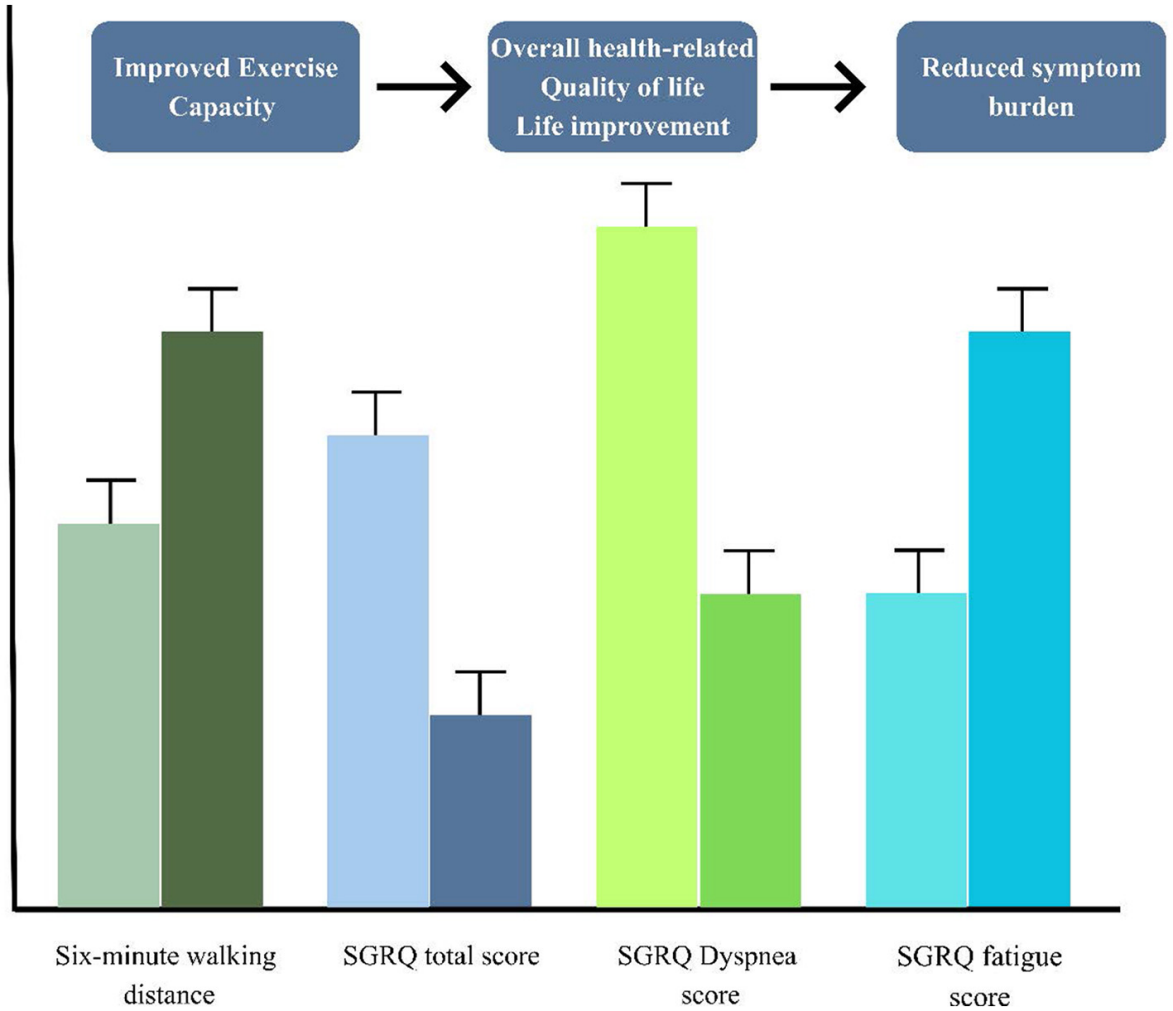
Changes in functional capacity and quality-of-life outcomes following pulmonary rehabilitation.

**Figure 5:**
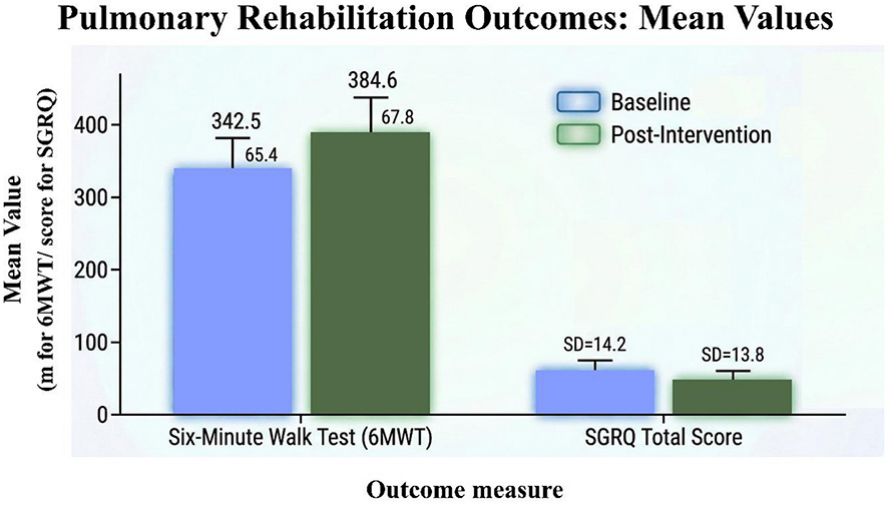
The bar graph illustrates the difference between baseline and post-intervention in the Six-Minute Walk Test (6MWT) distance and the total score of the St. George's Respiratory Questionnaire (SGRQ) after completion of a 3-month pulmonary rehabilitation program. The standard deviation is represented by error bars.

These are exploratory findings that indicate that participation in the pulmonary rehabilitation program was associated with improvements in both functional exercise capacity and patient reported quality of life, which highlight the possible benefit of a structured pulmonary rehabilitation intervention for improvements in objective and subjective outcomes. Nonetheless, the small number of people suggests that it is difficult to draw any definite conclusions. Figure 4 Summarize changes in objective (6MWD) and subjective (SGRQ) outcomes in a single visual. Figure 5 shows a comparative graphical analysis of baseline vs. post-intervention outcomes relating to functional capacity and health related quality of life.

### 3.4 Domain-specific analysis of SGRQ components

In-depth evaluation of the SGRQ symptom domain (Table 4) showed that most individual respiratory symptoms, including cough frequency, sputum, wheezing, and morning symptoms, did not show statistically significant changes post-intervention. This result aligns with the SGRQ symptom domain's construction. The domain is based on a 12-month recall interval and may be unresponsive to short-term treatments like a 3-month rehabilitation program. Interpretation of short-term changes in the SGRQ symptom domain should be made with caution because the questionnaire uses a 12-month recall period, which may limit its ability to detect symptom changes over a relatively short intervention period such as 3 months (*p* = 0.03). The analysis showed an increase in respiratory infections (*p* = 0.02) (30) was reported. However, considering the small sample size and the observational nature of the study, the rationale in the composition of this observation is indisputable from the data available. The recall of symptoms is conducted over a year and is not expected to change much during rehabilitation. Functional improvements in daily activities appeared early. Borderline improvements were recorded in breathlessness during indoor walking and bathing or showering (both *p* = 0.08) in the activity domain. However, most activity-related measures were not significant. This may be due to the small sample size and advanced disease in some participants.

**Table 4:** Summary of changes in SGRQ symptom components after intervention.

Symptom component	Pre-intervention (Mean ±SD)	Post-intervention (Mean ±SD)	Mean difference	*p-value*
Cough frequency (Almost every day – None)	38.7 ± 16.9	33.1 ± 15.8	−5.62	0.29
Sputum production frequency	44.8 ± 15.3	36.9 ± 13.6	−7.92	0.14
Shortness of breath frequency	23.1 ± 6.1	25.0 ± 6.4	1.93	0.32
Wheezing frequency	29.6 ± 12.8	30.2 ± 13.1	0.58	0.88
Severe respiratory attacks (More than 3 – None)	16.8 ± 8.7	26.1 ± 9.1	9.32	0.03
Duration of worst attack (A week or more – Less than a day)	36.5 ± 10.7	29.4 ± 11.2	−7.12	0.07
Good days in a typical week (No good days – Every day is good)	43.0 ± 19.9	38.2 ± 18.7	−4.79	0.41
Morning wheezing (Yes – No)	41.1 ± 12.5	33.6 ± 12.0	−7.48	0.25

Clinically meaningful and consistent improvements occurred in the impact domain. Greater changes were seen in breathlessness during speaking (*p* = 0.03), patient beliefs regarding progress of the disease (*p* = 0.00), perceptions of effort required for activities (*p* = 0.00), and side effects related to medication (*p* = 0.03). These results indicate that psychological wellbeing, disease perception, and functional confidence improved after pulmonary rehabilitation.

Analysis of the SGRQ symptom domain showed that most respiratory symptoms did not change after the intervention (31). The number of respiratory infections was the only symptom measured with statistical significance (*p* = 0.02). The observed change in reported respiratory infections should be interpreted cautiously. Given the limited sample size and exploratory design of this study, the data do not allow conclusions regarding the underlying causes of this observation. The SGRQ symptom question uses a year-long recollection period, so symptoms induced by a brief rehabilitation program are unlikely to change (32).

In the activity component, some daily activities showed borderline significance in the paired *t*-test. Breathlessness during indoor walking (*p* = 0.08) and during bathing or showering (*p* = 0.08) reached significance, while most activity-related measures did not change. The effect element showed more apparent benefits. Breathlessness during talking (*p* = 0.03), the belief that chest condition will not improve (*p* = 0.00), the perceived effort needed for tasks (*p* = 0.00), and some unpleasant medication side effects (*p* = 0.03) all showed significant improvement. Other impact measures did not show substantial post-measure changes.

### 3.5 Correlation analysis

**Figure 6:**
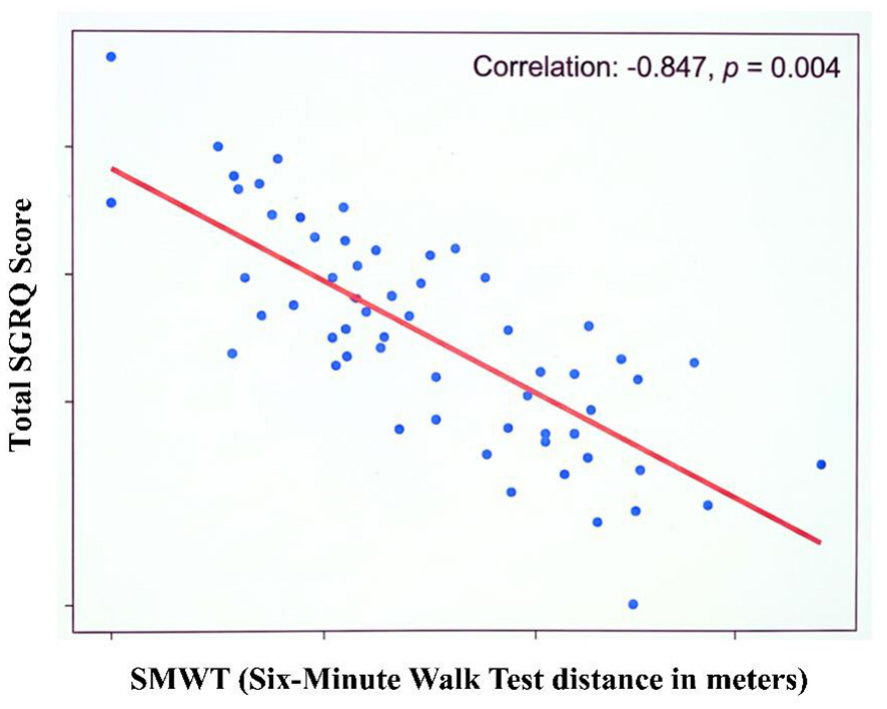
Scatter plot illustrating the relationship between changes in six-minute walk distance and changes in SGRQ total score following pulmonary rehabilitation.

Correlation analysis showed that there was a strong negative relationship between alteration in the 6MWT distance and alteration in the Total SGRQ score (*r* = −0.847, *p* = 0.004) as represented in Figure 6. This result indicates that as functional exercise capacity improved, participants experienced a greater reduction in perceived disease burden and an improvement in quality of life. Subgroup analyses by education level, occupational risk, and area of residence did not show any significant associations with the outcome measures.

The findings indicate that 3-month pulmonary rehabilitation is associated with meaningful improvements in exercise capacity, overall quality of life, and disease impact in patients with ILD. In comparison, individual symptom and activity measurements showed minor short-term changes. However, there were significant improvements in the impact domain and global outcome measures. Based on these exploratory findings, it can be postulated that pulmonary rehabilitation may result in measurable improvements in both the functional status of the patient, and also perceived quality of life; nevertheless, the small number of cases limits any definitive claims. They also provide helpful information about incorporating this intervention into multidisciplinary management.

## 4 Discussion

PR is now recognized as essential in the treatment of chronic respiratory disease. However, the best timing, duration, and form of PR for interstitial lung disease (ILD) are unclear (25) examined early PR in patients with fibrotic ILD (FILD). They found that those with low functional capacity and poor SGRQ scores gained the most in exercise ability. Despite subgroup improvements, overall, Six-Minute Walk Test (6MWT) distance and SGRQ scores did not change significantly after 6 weeks. In contrast, the present review found significant improvements in functional endurance and HRQOL. The mean change in 6MWD was 42.1 m, and the total SGRQ improved (*p* = 0.001), exceeding minimal clinically significant differences in ILD (21). Within the confines of this preliminary investigation, there is evidence to suggest that pulmonary rehabilitation is associated with tentative improvement in functional capacity and health-related quality of life; however, more extensive studies are imperative to provide more substantial evidence to substantiate these observations.

**Figure 7:**
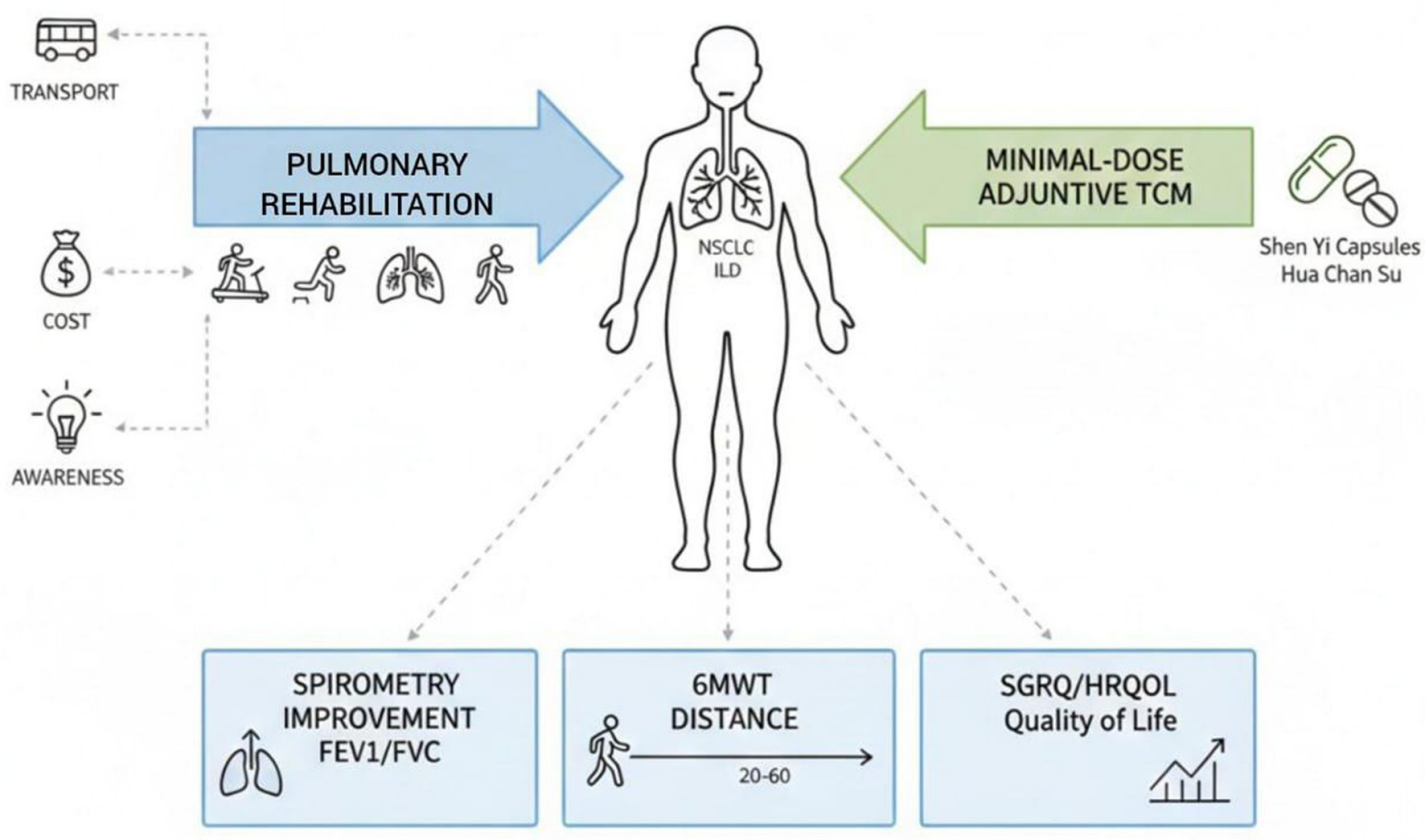
Conceptual illustration of the potential role of pulmonary rehabilitation in improving functional capacity and quality of life in patients with interstitial lung disease.

The importance of program structure and duration is shown in a randomized controlled trial by Márquez-Montes et al. (6). They tested a 6-month PR program in ILD patients. Their research found significant improvements in exercise tolerance, peripheral muscle strength, and HRQOL (35). The average 6MWD increased by 72 m after 6 months and remained stable at 1-year follow-up. SGRQ scores also improved, showing lasting benefits. Although their program lasted longer, our results show that significant gains can be achieved in as little as 3 months when PR is appropriately designed and tailored. For health systems with limited resources, offering long-term programs to many patients may not be possible (Figure 7).

A further link to existing literature is seen in the analysis of HRQOL domains (33). The most significant gains appeared in dyspnea-related and impact domains. This matches earlier studies that identified breathlessness as the most sensitive indicator of PR in ILD (26). Evidence suggests a statistically significant improvement in SGRQ scores in several areas, thereby suggesting improvements in perceived respiratory symptoms, limitations to functional activity and general quality of life following pulmonary rehabilitation. The SGRQ symptom and activity domains did not change significantly in the short term, likely due to the questionnaire's 12-month recall limit. Yet, the impact domain, which includes psychological burden, disease perception, and social functioning, showed notable improvement. These findings highlight that PR boosts functional confidence and coping more than it immediately alters chronic symptoms (27, 37).

Overall, current evidence shows that PR consistently improves functional capacity and HRQOL in ILD patients, regardless of program duration (34). Shorter programs offer quick benefits, while longer ones help maintain gains (28). The strong inverse relationship between improved 6MWD and lower SGRQ scores underscores the close link between physical performance and perceived disease burden. These findings, obtained from a small-scale preliminary cohort study, suggest that there may be potential advantages of pulmonary rehabilitation in improving functional capacity and quality of life in persons with interstitial lung disease. Nevertheless, more extensive, well-controlled studies are essential to prove these promising early observations (36).

However, knowledge gaps remain. Future research should study larger, multi-centered cohorts with extended follow-up. This would help identify the best duration, intensity, and maintenance strategies for PR. It would also help pinpoint which patient subgroups benefit most, based on subtype, impairment, or comorbidity. This evidence can improve guidelines and encourage PR as a standard in respiratory care (29).

## 5 Conclusion and future recommendations

### 5.1 Conclusion

The present investigation attempted to evaluate the potential impact of the structured pulmonary rehabilitation programme on functional capacity and health-related quality of life in patients with interstitial lung disease. Significant improvements in six-minute walk distance with decreases in scores on the St. George's Respiratory Questionnaire were seen after the 3-month intervention. Nonetheless, given the small sample size and the exploratory nature of the study, these results should be considered preliminary observations rather than conclusive evidence. Robust, adequately powered controlled trials are needed to support the efficacy of pulmonary rehabilitation interventions in this population of patients. The synthesized evidence showed consistent improvements in objective functional outcomes, especially in six-minute walk Test (6MWT). Significant decrements in St. George's Respiratory Questionnaire scores were observed after pulmonary rehabilitation, hence suggesting significant improvements in patient-reported quality of life.

These overlapping conclusions demonstrate that, in addition to improving physiological performance, PR positively affects patients' perception of wellbeing, functional confidence, and disease burden. Although these advantages are demonstrated, the implementation of PR in the real-world setting of ILD remains constrained by persistent, intertwined barriers. Patients lack awareness of ILD and its chronic effects. Many do not understand the possible benefits of rehabilitation. Transportation barriers, financial limitations, and the insufficient number of specialized rehabilitation facilities also hinder participation and compliance. These issues are especially acute in low- and middle-income environments, where healthcare and rehabilitation facilities at the community level are often inadequate. These obstacles highlight the need to expand alternative delivery models, such as telepulmonary rehabilitation (tele-PR) and community- or home-based PR programs. These models would improve accessibility, reduce economic load, and encourage long-term attendance. In the future, PR in ILD needs to become more personalized, adaptable, and community-based. Developing rehabilitation interventions based on disease severity, functional status, and local healthcare availability could maximize therapeutic effect and enhance uptake. Including PR in wider community health rehabilitation models can expand coverage of benefits beyond tertiary care. It can also help with long-term disease management. Future research should verify scalable PR delivery models, identify which patient groups benefit most, and develop evidence-based guidelines for integrating PR into ILD care pathways regularly.

### 5.2 Limitations

Several limitations affect the interpretation of the findings from this review. Most importantly, the small size of the quasi-experimental evidence reduces statistical power. This makes it impossible to draw conclusive results about the extent and consistency of pulmonary rehabilitation (PR) benefits for all people with interstitial lung disease (ILD). The short follow-up period is also a limitation. It prevents the determination of the long-term sustainability of functional and quality-of-life improvements from rehabilitation. For this reason, whether these benefits last over time after the intervention remains unclear.

Recruitment issues are also a limitation. The study population came from a highly specialized hospital, which narrows the pool of participants and introduces selection bias. Patients in these centers often differ in disease severity, access to care, and motivation compared to those in general or civilian hospitals. Their experiences may not represent the broader ILD population, especially people in rural or underserved areas.

A single-center design limits the external validity and applicability of these findings. Results may not apply to other health systems, regions, or clinical settings. Differences in rehabilitation facilities, staff experience, or patient service models can affect outcomes and feasibility. The lack of longitudinal follow-up also blocks the study of the long-term effects on physiological health and quality of life. Future studies should use multiple centers, larger sample sizes, and longer follow-ups. This will help confirm current findings and inform evidence-based recommendations for long-term PR in ILD treatment.

Another limitation of the present study is the small sample size, which is a direct result of the exploratory and feasibility-oriented aims defining the present study. In the absence of a formal power calculation, the study was not designed to test specific hypotheses or detect small effect sizes. Accordingly, the results should be interpreted as preliminary observations that provide possible guidance for planning larger, adequately powered investigations of the effect of pulmonary rehabilitation in patients with interstitial lung disease.

Another limitation relates to the use of the St. Georges Respiratory Questionnaire (SGRQ) to evaluate the change in symptoms. Although the SGRQ is commonly used to assess health related quality of life in chronic respiratory diseases, the symptom domain uses a 12 month recall period, which might make it less sensitive for detecting short term changes in response to a 3 month pulmonary rehabilitation intervention; hence improvements noted on symptom related measures should be interpreted cautiously.

Future studies examining pulmonary rehabilitation in interstitial lung disease might benefit from the use of other types of patient-reported outcome measures that have been found to be more sensitive to short-term clinical changes.

### 5.3 Recommendations

Future studies of pulmonary rehabilitation (PR) in interstitial lung disease (ILD) must focus on larger and more diverse samples. This will enhance statistical power and externality between ILD subtypes. Expanding recruitment to other age groups, disease severity levels, and sociodemographic groups would enable meaningful subgroup analysis. It would also support evidence-based personalization of rehabilitation in each case. Moreover, a longer follow-up is needed to evaluate the sustainability of functional and quality-of-life improvements. Such studies can identify whether maintenance or booster rehabilitation sessions help support long-term results. It is suggested to use multicenter and, where possible, multinational designs. This would help capture variations in health care delivery and rehabilitation infrastructure. It would also improve access to patients across settings and aid translation of results into clinical practice. Additional research should incorporate multidimensional outcome measures, including mental health, fatigue, physical activity, and patient-reported outcomes. Future studies should focus on identifying patient subgroups most likely to respond to PR. Subgrouping can be based on ILD subtype, baseline functional status, symptom burden, and comorbidities. Such a strategy would help build tailored rehab programs, streamline resource distribution, and enhance patient-centered care for ILD management.

## Data Availability

The raw data supporting the conclusions of this article will be made available by the authors, without undue reservation.
